# Columella’s Proposition

**Published:** 1902-03

**Authors:** 


### COLUMELLA'S PROPOSITION.

We print in this number a communication from Prof. Law, bearing upon the proposition made in our pages as to a better method of dealing with bovine tuberculosis. Several salient features are presented, all of which should be most earnestly discussed that a more satisfactory plan may be evolved and a broader protection guaranteed of all the diverse interests involved. Several other communications will appear in the April number, and we trust that any of our readers who are dealing with this subject will feel free to contribute any news or light on this topic.

